# Efficient learning of non-autoregressive graph variational autoencoders for molecular graph generation

**DOI:** 10.1186/s13321-019-0396-x

**Published:** 2019-11-21

**Authors:** Youngchun Kwon, Jiho Yoo, Youn-Suk Choi, Won-Joon Son, Dongseon Lee, Seokho Kang

**Affiliations:** 1 Samsung Advanced Institute of Technology, Samsung Electronics Co. Ltd., 130 Samsung-ro, Yeongtong-gu, Suwon, Republic of Korea; 2 Department of Computer Science and Engineering, Seoul National University, 1 Gwanak-ro, Gwanak-gu, Seoul, Republic of Korea; 3 Department of Systems Management Engineering, Sungkyunkwan University, 2066 Seobu-ro, Jangan-gu, Suwon, Republic of Korea

**Keywords:** Molecular graph, Variational autoencoder, Graph neural network, Deep learning

## Abstract

With the advancements in deep learning, deep generative models combined with graph neural networks have been successfully employed for data-driven molecular graph generation. Early methods based on the non-autoregressive approach have been effective in generating molecular graphs quickly and efficiently but have suffered from low performance. In this paper, we present an improved learning method involving a graph variational autoencoder for efficient molecular graph generation in a non-autoregressive manner. We introduce three additional learning objectives and incorporate them into the training of the model: approximate graph matching, reinforcement learning, and auxiliary property prediction. We demonstrate the effectiveness of the proposed method by evaluating it for molecular graph generation tasks using QM9 and ZINC datasets. The model generates molecular graphs with high chemical validity and diversity compared with existing non-autoregressive methods. It can also conditionally generate molecular graphs satisfying various target conditions.

## Introduction

In recent years, machine learning has been actively adopted to accelerate the discovery of novel molecules with desired properties [1–4]. While traditional approaches, such as manual design and enumeration [5–8], depend highly on domain knowledge and intuition of human experts, machine learning approaches have allowed automated design of desired molecules in a data-driven manner. Thus, they have attracted considerable attention from academia and industry. A typical way to accomplish so is to construct a generative model by learning from data to capture the underlying distribution of the data, and use it to generate new molecules [9, 10]. The large number of known molecules that have been discovered in the past can be a valuable source for the data [11, 12]. Although this research area is currently in the incipient stage and remains challenging, studies are increasingly performed, demonstrating improved effectiveness.

The main consideration is how to represent molecules to be processed within a model and to be generated by a model. Various types of molecular representations can be considered. Among them, most existing studies have employed a simplified molecular-input line-entry system (SMILES) [13] to represent molecules. SMILES is a line notation that encodes its graph structure via depth-first traversal into a sequence of symbols with a simple vocabulary and grammar rules. There have been implemented deep generative models based on a recurrent neural network (RNN), which learn a generative distribution from data to produce a SMILES string from a latent vector. They include RNN language models [14–17], variational autoencoders (VAEs) [18–21] and generative adversarial networks (GANs) [22, 23]. To conditionally generate SMILES strings with specified target properties, additional optimization procedures have been adopted along with the generative models, including Bayesian optimization [18] and reinforcement learning [14, 15, 22, 23]. Conditional generative models, which directly sample new SMILES strings from a conditional generative distribution without any extra optimization procedure, have also been presented for targeted generation [19, 24].

SMILES representation has proven useful in learning generative models for molecular design tasks owing to its simplicity. However, several limitations hinder the generation of chemically valid molecules. This representation cannot fully represent the entire chemical space, but only a part of the chemical space can be represented as SMILES strings. It is inadequate to capture the similarity between molecules, because a small perturbation in a molecule may result in a significant change in the representation. To address such limitations, numerous research attempts have been made to generate molecular graphs directly using generative models. A molecular graph ${\mathcal {G}}$ is a natural representation of a molecule, which is expressed as an undirected graph of varying size whose nodes and edges correspond to atoms and bonds, respectively. This representation provides high coverage of the chemical space, and can convey various chemical features within it.

With recent advances in graph neural networks [25–27], various methods for extending deep generative models to molecular graph generation have been proposed. They can be categorized into two main approaches: non-autoregressive and autoregressive. The non-autoregressive approach allows more principled use of generative models, generating a molecular graph ${\mathcal {G}}$ directly from a latent vector ${\mathbf {z}}$ using a non-autoregressive distribution $p({\mathcal {G}}|{\mathbf {z}})$ without any iterative procedure. Few methods based on this approach have been presented, owing to the challenge imposed by graph isomorphism, meaning that a molecular graph is invariant to permutations of its nodes. Notably, Simonovsky and Komodakis [28] presented a non-autoregressive VAE that generates molecular graphs, named GraphVAE. Its training requires calculating the reconstruction loss for generated graphs. The problem of graph isomorphism is addressed by a graph matching procedure that entails expensive computation. De Cao and Kipf [29] sidestepped graph isomorphism by using a non-autoregressive GAN for molecular graph generation, named MolGAN. Its training however suffers from the mode-collapse problem, thus less diverse molecular graphs are generated. The non-autoregressive approach successfully generates small molecular graphs in a very fast and efficient manner but suffers from difficulty in model training and a low validity ratio when generating larger graphs. Owing to such limitations, the autoregressive approach, which aims at sequentially generating a molecular graph node by node using an autoregressive distribution, has been a main research direction [30–33]. These methods successfully generate molecular graphs with a high validity ratio, while involves an iterative procedure for each generation which makes them less efficient.

This work focuses on the non-autoregressive approach for fast and efficient generation of molecular graphs. Here we propose an efficient learning method to train a VAE that generates molecular graphs in a non-autoregressive manner. To improve the molecular graph generation, we train the VAE by incorporating three additional learning objectives: approximate graph matching, reinforcement learning, and auxiliary property prediction. The trained VAE can almost always generate chemically valid molecular graphs with improved diversity compared with existing non-autoregressive methods. The proposed method also allows constrained generation of molecular graphs with specified target properties.

## Methods

### Molecules as graphs

In this work, we use the molecular graph representation defined as follows. A molecule is represented by an undirected graph ${\mathcal {G}}=({\mathcal {V}},{\mathcal {E}})$ with up to *m* nodes, where ${\mathcal {V}}$ and ${\mathcal {E}}$ represent the set of nodes and the set of edges, respectively. The node vectors ${\mathbf {v}}^i \in {\mathcal {V}}$ and edge vectors ${\mathbf {e}}^{i,j} \in {\mathcal {E}}$ are associated with heavy atoms and their bonds, respectively, in the molecule. It should be noted that ${\mathbf {e}}^{i,j}={\mathbf {e}}^{j,i}$ because we use an undirected graph. For the *i*-th atom, ${\mathbf {v}}^i=(v^{i,1},\ldots ,v^{i,p})$ is a *p*-dimensional vector formed by concatenating three one-hot vectors indicating the atom type, formal charge, and number of explicit hydrogens. The dimension *p* depends on the dataset used. For the bond between the *i*-th and *j*-th atoms, ${\mathbf {e}}^{i,j}=(e^{i,j,1},\ldots ,e^{i,j,q})$ is a *q*-dimensional one-hot vector associated with the bond type. We kekulize the molecule for simplicity so that the only bond types to consider are single, double, triple, and none, hence $q=4$. Additionally, the properties of the molecule are represented as a property vector ${\mathbf {y}}=(y^1,\ldots ,y^l)$.

### Graph variational autoencoder

We construct a conditional version of the graph VAE [34] in a non-autoregressive manner. It seeks to find the generative distribution of ${\mathcal {G}}$ conditioned on a latent vector ${\mathbf {z}}$ and a property vector ${\mathbf {y}}$ and parameterized by $\theta$, denoted as $p_\theta ({\mathcal {G}}|{\mathbf {z}},{\mathbf {y}})$. The prior distributions of ${\mathbf {z}}$ and ${\mathbf {y}}$ are assumed to be $p({\mathbf {z}})={\mathcal {N}}({\mathbf {z}}|{\mathbf {0}},{\mathbf {I}})$ and $p({\mathbf {y}})={\mathcal {N}}({\mathbf {y}}|\varvec{\mu }_{\mathbf {y}},\varvec{\Sigma }_{\mathbf {y}})$, respectively. To address the intractability of the posterior distribution $p_\theta ({\mathbf {z}}|{\mathcal {G}},{\mathbf {y}})$, we introduce an approximate posterior distribution $q_\phi ({\mathbf {z}}|{\mathcal {G}},{\mathbf {y}})={\mathcal {N}}({\mathbf {z}}|{\varvec{\mu }_{\mathbf {z}}({\mathcal {G}},{\mathbf {y}}), \text {diag}(\varvec{\sigma }^2_{\mathbf {z}}({\mathcal {G}},{\mathbf {y}})}))$, which has a normal distribution and is parameterized by $\phi$.

The distributions $q_\phi ({\mathbf {z}}|{\mathcal {G}},{\mathbf {y}})$ and $p_\theta ({\mathcal {G}}|{\mathbf {z}},{\mathbf {y}})$ are called the encoder and decoder of the VAE, respectively. For the encoder, we use a message passing neural network (MPNN) [27], which is a variant of a graph neural network that operates directly on graphs of different sizes and is invariant to graph isomorphism. The encoder takes ${\mathcal {G}}$ and ${\mathbf {y}}$ and outputs the mean vector $\varvec{\mu }_{\mathbf {z}}({\mathcal {G}},{\mathbf {y}})$ and variance vector $\varvec{\sigma }^2_{\mathbf {z}}({\mathcal {G}},{\mathbf {y}})$, from which ${\mathbf {z}}$ is sampled based on reparametrization as $\varvec{\mu }_{\mathbf {z}}({\mathcal {G}},{\mathbf {y}})+\varvec{\epsilon } \odot \varvec{\sigma }^2_{\mathbf {z}}({\mathcal {G}},{\mathbf {y}})$ with $\varvec{\epsilon } \sim {\mathcal {N}}({\mathbf {0}},{\mathbf {I}})$. The decoder is modeled as a fully-connected neural network that outputs $mp+m(m-1)q/2$ values at once from ${\mathbf {z}}$ and ${\mathbf {y}}$ with node-wise and edge-wise softmax activation. The output values form a probabilistic graph $g({\mathbf {z}},{\mathbf {y}})$ composed of *m* nodes and $m(m-1)/2$ edges.

The original learning objective of the VAE is given with respect the parameters $\phi$ and $\theta$ as:

1 $$\begin{aligned} {\mathcal {L}}_\text {VAE} (\phi ,\theta ) = {\mathbb {E}}_{{\mathbf {z}} \sim q_\phi ({\mathbf {z}}|{\mathcal {G}},{\mathbf {y}})} \left[ -\log p_\theta ({\mathcal {G}}|{\mathbf {z}},{\mathbf {y}}) \right] + {\mathcal {D}}_\text {KL}(q_\phi ({\mathbf {z}}|{\mathcal {G}},{\mathbf {y}})|| p({\mathbf {z}}) ), \end{aligned}$$

where the first and second terms on the right-hand side are regarded as the reconstruction loss and regularization loss, respectively. Owing to graph isomorphism, the calculation of the reconstruction loss necessitates a graph matching procedure that involves comparing an input graph and its probabilistic reconstruction which is computationally expensive. For example, the max-pooling matching algorithm has computational complexity of ${\mathcal {O}}(m^4)$ [28].

To make the learning more efficient, we introduce an approximate graph matching procedure, which aims to alleviate the computational burden for the reconstruction loss. Additionally, we incorporate reinforcement learning and auxiliary property prediction into the training of the VAE to further improve the generation performance. Details regarding the learning objectives utilized in this work are presented in the following subsection.

### Approximate graph matching

The reconstruction loss ${\mathbb {E}}_{{\mathbf {z}} \sim q_\phi ({\mathbf {z}}|{\mathcal {G}},{\mathbf {y}})} \left[ -\log p_\theta ({\mathcal {G}}|{\mathbf {z}},{\mathbf {y}}) \right]$ involves comparing an original input graph ${\mathcal {G}}=({\mathcal {V}},{\mathcal {E}})$ and its reconstruction by the VAE. In this subsection, we denote the reconstruction of ${\mathcal {G}}$ as a probabilistic graph $\widetilde{{\mathcal {G}}}=(\widetilde{{\mathcal {V}}},\widetilde{{\mathcal {E}}})$, where $\widetilde{{\mathbf {v}}}^i \in \widetilde{{\mathcal {V}}}$ and $\widetilde{{\mathbf {e}}}^{i,j} \in \widetilde{{\mathcal {E}}}$. Because the reconstruction loss must be invariant to graph isomorphism, a graph matching procedure that seeks the best possible matching between the two graphs is needed. To avoid expensive computation, we propose to use approximate graph matching. The main idea is to approximate the distance between ${\mathcal {G}}$ and $\widetilde{{\mathcal {G}}}$ by comparing the numbers of atom types, bond types, atom-bond pair types, and atom-bond-atom pair types.

Assuming that each edge vector is represented as a four-dimensional vector as ${\mathbf {e}}^{i,j}=(e^{i,j(\texttt {single})},e^{i,j(\texttt {double})},e^{i,j(\texttt {triple})},e^{i,j(\texttt {none})})$, the reconstruction loss is approximated as follows:

2 $$\begin{aligned}&{\mathbb {E}}_{{\mathbf {z}} \sim q_\phi ({\mathbf {z}}|{\mathcal {G}},{\mathbf {y}})} \left[ -\log p_\theta ({\mathcal {G}}|{\mathbf {z}},{\mathbf {y}}) \right] \simeq \left\| \left( \sum _i{{\mathbf {v}}^i}\right) - \left( \sum _i{\widetilde{{\mathbf {v}}}^i}\right) \right\| ^2 \nonumber \\&\quad + \left\| \left( \sum _{i,j}{{\mathbf {e}}^{i,j}}\right) - \left( \sum _{i,j}{\tilde{{\mathbf {e}}}^{i,j}}\right) \right\| ^2\nonumber \\&\quad + \sum _{\texttt {b}\in {\{\texttt {single},\texttt {double},\texttt {triple}\}}} \left\| \left( \sum _{i,j}{e^{i,j (\texttt {b})}{\mathbf {v}}^i }\right) - \left( \sum _{i,j}{{\tilde{e}}^{i,j (\texttt {b})}\mathbf {{\widetilde{v}}}^i }\right) \right\| ^2\nonumber \\&\quad + \sum _{\texttt {b}\in {\{\texttt {single},\texttt {double},\texttt {triple}\}}} \left\| \left( \sum _{i,j}{e^{i,j (\texttt {b})}{\mathbf {v}}^i {{\mathbf {v}}^{jT}}}\right) - \left( \sum _{i,j}{ {\tilde{e}}^{i,j (\texttt {b})}\mathbf {{\widetilde{v}}}^i {\mathbf {{\widetilde{v}}}^{jT}}}\right) \right\| ^2, \end{aligned}$$

where ${{\mathbf {v}}}^i \in {{\mathcal {V}}}$, ${{\mathbf {e}}}^{i,j} \in {{\mathcal {E}}}$, $\widetilde{{\mathbf {v}}}^i \in \widetilde{{\mathcal {V}}}$, and $\widetilde{{\mathbf {e}}}^{i,j} \in \widetilde{{\mathcal {E}}}$. When calculating the approximated reconstruction loss, we discard the non-atom and non-bond types from the vectors. The first, second, third, and fourth terms on the right-hand side correspond to the comparison of the numbers of atom types, bond types, atom-bond pair types, and atom-bond-atom pair types, respectively. They are independent of node ordering because they summate over the nodes in a graph, and are thus invariant to graph isomorphism. All the operations in the above equation are differentiable.

### Reinforcement learning

We further improve the VAE via reinforcement learning with the aim of generating chemically valid molecules. We adopt a deterministic policy gradient framework, in which the decoder of the VAE is regarded as a policy network that takes the two vectors ${\mathbf {z}}$ and ${\mathbf {y}}$ as state inputs. It outputs a probabilistic graph as an action from the state. The reward for the action is the chemical validity of the probabilistic graph, which is evaluated using an external reward function *R*. The reward function returns 1 if the probabilistic graph can be decoded into a chemically valid molecular graph and 0 otherwise. In this work, the chemical validity of a molecular graph ${\mathcal {G}}$ is evaluated via a sanitization check. With the reward function, the policy network learns how to generate a probabilistic graph that fulfills the expected reward of 1.

We wish to optimize the molecular graph generation of the VAE for maximizing the external reward function *R*. Because the reward function is non-differentiable, it cannot be incorporated directly into the learning procedure. We build a reward network *r* that approximates the reward function *R*. The reward network is modeled as an MPNN with a sigmoid output. It takes a probabilistic graph as input and predicts its actual reward value. The reward network can backpropagate the VAE. We train the VAE to generate a probabilistic graph towards maximizing the output of the reward network.

For the learning objective, we derive two additional losses ${\mathcal {L}}_\text {RL} (\phi ,\theta )$ and ${{\mathcal {L}}_\text {RL} (r)}$ as:

3 $$\begin{aligned} {{\mathcal {L}}_\text {RL} (\phi ,\theta )} & =  {} {\mathbb {E}}_{{\mathbf {z}} \sim q_\phi ({\mathbf {z}}|{\mathcal {G}},{\mathbf {y}})}{\mathbb {E}}_{{\mathcal {G}} \sim p_\theta ({\mathcal {G}}|{\mathbf {z}},{\mathbf {y}})}{[-\log r({\mathcal {G}}) ]} \nonumber \\ & \quad + {\mathbb {E}}_{{\mathbf {z}} \sim p({\mathbf {z}}), {\mathbf {y}} \sim p({\mathbf {y}})}{\mathbb {E}}_{{\mathcal {G}} \sim p_\theta ({\mathcal {G}}|{\mathbf {z}},{\mathbf {y}})}{[-\log r({\mathcal {G}})]}; \end{aligned}$$

4 $$\begin{aligned} {{\mathcal {L}}_\text {RL} (r)} &=  {} {-R({\mathcal {G}})\log r({\mathcal {G}})-(1-R({\mathcal {G}}))\log (1-r({\mathcal {G}}))}\nonumber \\ & \quad +{\mathbb {E}}_{{\mathbf {z}} \sim q_\phi ({\mathbf {z}}|{\mathcal {G}},{\mathbf {y}})}{\mathbb {E}}_{{\mathcal {G}} \sim p_\theta ({\mathcal {G}}|{\mathbf {z}},{\mathbf {y}})}{[- R({\mathcal {G}}) \log r({\mathcal {G}}) - (1-R({\mathcal {G}})) \log (1-r({\mathcal {G}})) ]}\nonumber \\ & \quad + {\mathbb {E}}_{{\mathbf {z}} \sim p({\mathbf {z}}), {\mathbf {y}} \sim p({\mathbf {y}})}{\mathbb {E}}_{{\mathcal {G}} \sim p_\theta ({\mathcal {G}}|{\mathbf {z}},{\mathbf {y}})}{[- R({\mathcal {G}}) \log r({\mathcal {G}}) - (1-R({\mathcal {G}})) \log (1-r({\mathcal {G}})) ]}. \end{aligned}$$

The VAE is trained for minimizing ${\mathcal {L}}_\text {RL} (\phi ,\theta )$, while the reward network *r* is trained to minimize ${{\mathcal {L}}_\text {RL} (r)}$.

It should be noted that we can impose extra domain-specific constraints regarding structures or properties on the external reward function *R* for constrained generation. For example, we can define a blacklist of undesired substructures and make the value of the reward function 0 when its input contains any substructure in the blacklist. This prevents generated molecules from having undesired substructures.

### Auxiliary property prediction

Augmenting a generative model with side information has known to improve the quality of generated samples as well as the stability of model training [19, 35]. We incorporate auxiliary property prediction into the VAE learning to enable generating probabilistic graphs that correspond to desired properties as well as to diversify the generated outcomes. We build a predictor network as an MPNN with *l* linear outputs. It learns from the training dataset to predict the property vector ${\mathbf {y}}$ of a given graph. Because the predictor network can backpropagate the VAE, we train the VAE to generate a probabilistic graph whose corresponding ${\mathbf {y}}$ is to be reconstructed by the predictor network.

For learning with auxiliary property prediction, we derive two additional losses ${\mathcal {L}}_\text {Y} (\phi ,\theta )$ and ${{\mathcal {L}}_\text {Y} (f)}$ as:

5 $$\begin{aligned} {\mathcal {L}}_\text {Y} (\phi ,\theta ) & =  {} {\mathbb {E}}_{{\mathbf {z}} \sim q_\phi ({\mathbf {z}}|{\mathcal {G}},{\mathbf {y}})}{\mathbb {E}}_{{\mathcal {G}} \sim p_\theta ({\mathcal {G}}|{\mathbf {z}},{\mathbf {y}})}{ \left[ R({\mathcal {G}}) \cdot ||{\mathbf {y}}-f({\mathcal {G}})||^2 \right] }\nonumber \\ & \quad + {\mathbb {E}}_{{\mathbf {z}} \sim p({\mathbf {z}}), {\mathbf {y}} \sim p({\mathbf {y}})}{\mathbb {E}}_{{\mathcal {G}} \sim p_\theta ({\mathcal {G}}|{\mathbf {z}},{\mathbf {y}})}{\left[ R({\mathcal {G}}) \cdot ||{\mathbf {y}}-f({\mathcal {G}})||^2 \right] }; \end{aligned}$$

6 $$\begin{aligned} {\mathcal {L}}_\text {Y} (f)= & {} ||{\mathbf {y}}-f({\mathcal {G}})||^2. \end{aligned}$$

Only the probabilistic graphs that are deemed valid by the external reward function *R* are incorporated into the first loss ${\mathcal {L}}_\text {Y} (\phi ,\theta )$. The VAE is trained to minimize ${\mathcal {L}}_\text {Y} (\phi ,\theta )$. Simultaneously, the predictor network *f* is trained to minimize ${{\mathcal {L}}_\text {Y} (f)}$.

### Learning from data

The proposed model is composed of four main components: the encoder network $q_\phi$, decoder network $p_\theta$, reward network *r*, and predictor network *f*. The full learning objective combines the vanilla objective of VAE (1) along with objectives for approximate graph matching (2), reinforcement learning (3–4), and auxiliary property prediction (5–6). The objective functions for the VAE part and the other part are ${\mathcal {J}}_1$ and ${\mathcal {J}}_2$, respectively, given as:

7 $$\begin{aligned} {\mathcal {J}}_1 (\phi ,\theta )= & {} \sum _{({\mathcal {G}}_t,{\mathbf {y}}_t)\sim {p}_\text {data}}\left[ {\mathcal {L}}_\text {VAE} (\phi ,\theta )+ \beta _1 \cdot {\mathcal {L}}_\text {RL} (\phi ,\theta )+ \beta _2 \cdot {\mathcal {L}}_\text {Y} (\phi ,\theta )\right] ; \end{aligned}$$

8 $$\begin{aligned} {\mathcal {J}}_2 (r,f)= & {} \sum _{({\mathcal {G}}_t,{\mathbf {y}}_t)\sim {p}_\text {data}}\left[ \beta _1 \cdot {\mathcal {L}}_\text {RL} (r)+ \beta _2 \cdot {\mathcal {L}}_\text {Y} (f)\right] , \end{aligned}$$

where $\beta _1$ and $\beta _2$ are hyperparameters that control the trade-off between different learning objectives.

Given an empirical data distribution $p_\text {data}({\mathcal {G}},{\mathbf {y}})$, we train the entire model for minimizing the two objective functions ${\mathcal {J}}_1(\phi , \theta )$ and ${\mathcal {J}}_2(r,f)$ simultaneously. For each iteration, a training batch *X* is sampled from the data distribution. The VAE parameters $\phi$ and $\theta$ are updated via gradient descent of ${\mathcal {J}}_1(\phi , \theta )$ on *X*, and the reward network *r* and predictor network *f* are updated via gradient descent of ${\mathcal {J}}_2(r,f)$. Algorithm 1 presents the pseudocode of the learning procedure. 
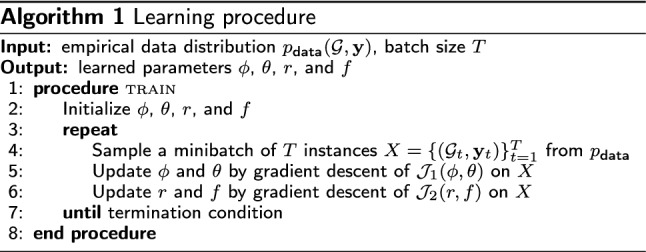


### Molecular graph generation

Once the model is trained, we use the decoder $p_\theta ({\mathcal {G}}|{\mathbf {z}},{\mathbf {y}})$ to generate molecular graphs. For unconditional generation, ${\mathbf {y}}_*$ is sampled from its prior distribution $p({\mathbf {y}})$. To conditionally generate molecular graphs, ${\mathbf {y}}_*$ is sampled from a conditional distribution. For example, if the target condition is given as $y^k=\tau$, then ${\mathbf {y}}_* \sim p({\mathbf {y}}|y^k=\tau )$. We sample ${\mathbf {z}}_*$ from $p({\mathbf {z}})$. Given ${\mathbf {y}}_*$ and ${\mathbf {z}}_*$, the decoder returns a probabilistic output, which is decoded based on node-wise and edge-wise argmax to obtain a molecular graph ${\mathcal {G}}_*$ as

9 $$\begin{aligned} {\mathcal {G}}_* =\underset{{\mathcal {G}}}{\text {argmax }}p_\theta ({\mathcal {G}}|{\mathbf {z}}={\mathbf {z}}_*,{\mathbf {y}}={\mathbf {y}}_*). \end{aligned}$$

We use a simple decoding method to discretize probabilistic outputs for deriving molecular graphs. Some studies reported that post-processing of probabilistic outputs based on such methods as maximum spanning tree [28] and beam search [36] can improve the validity of the generated molecular graphs.

## Results and discussion

### Datasets

We conducted experiments to investigate the effectiveness of the proposed method. In this work, we utilized two molecular datasets: QM9 and ZINC. They are publicly available and have commonly been used for the evaluation of molecular graph generation.

The QM9 dataset [37, 38] originally contains 133,885 organic molecules with at most nine heavy atoms of the following types: {C, N, O, F, none}. We sampled 100,000 unique molecular graphs from the original set. For each atom in a molecular graph, the formal charge and the number of explicit hydrogens belonged to $\{-\,1,0,1\}$ and $\{0,1,2,3\}$, respectively. Thus, the dimensions *p* and *q* were 12 and 4, respectively.

The ZINC dataset was constituted by sampling 100,000 unique molecular graphs from the drug-like part of the ZINC database [11]. Each molecular graph in the dataset contained up to 38 heavy atoms with ten atom types: {C, N, O, F, P, S, Cl, Br, I, none}. In the dataset used, the formal charge and the number of explicit hydrogens were in $\{-\,1,0,1\}$ and $\{0,1,2,3\}$, respectively. The dimensions *p* and *q* were 17 and 4, respectively.

We employed two properties that are readily calculable using the RDKit package [39]: the molecular weight (MolWt) and Wildman-Crippen partition coefficient (LogP) [40]. The property vector of each molecular graph was constituted with the three properties. Thus, the dimension *l* was 2. These properties can be evaluated efficiently without high costs for newly generated molecular graphs.

### Experiments

**Fig. 1:**
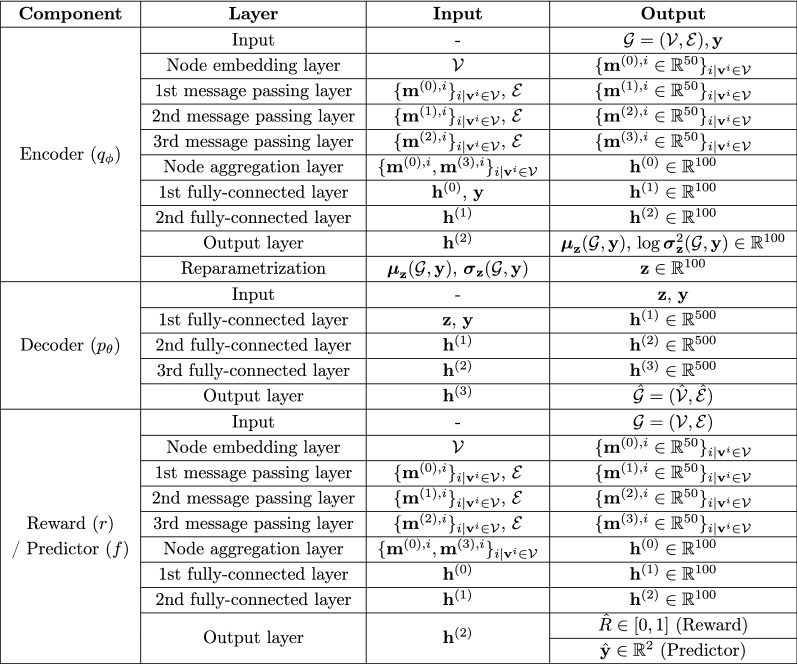
Model architecture used in case study

In the proposed method, the model architecture was determined as follows. For the molecular graph representation, the hyperparameter *m* was set as 5 plus the maximum number of heavy atoms in a molecule in the dataset used, i.e., *m* = 14 for QM9 and *m* = 43 for ZINC. The encoder, reward, and predictor networks were modeled as MPNNs. Each MPNN had three message passing layers with 50 hidden units, followed by a 100-dimensional node aggregation layer, which was then further processed by two fully-connected layers with 100 tanh hidden units. The decoder network was a fully-connected network with three fully-connected layers having dimensions of 500 with LeakyReLU activation. The dimension of ${\mathbf {z}}$ was set as 100. Each property in ${\mathbf {y}}$ was normalized to have a mean of 0 and a standard deviation of 1 for training. For the prior distribution $p({\mathbf {y}})$, we estimated the mean vector $\hat{\varvec{\mu }}_{\mathbf {y}}$ and covariance matrix $\hat{\varvec{\Sigma }}_{\mathbf {y}}$ from the training dataset. For reinforcement learning, the external reward function was implemented using the RDKit package [39]. Figure 1 shows the architectural details of the model used in the experiments. For the objective functions, the hyperparameters $\beta _1$ and $\beta _2$ were both set as 1. Given a dataset, the model was trained for 50 epochs using the RMSProp optimizer with a learning rate of 0.0005 and a batch size of 20.

We compared the proposed method with two baseline methods that generate molecular graphs in a non-autoregressive manner: GraphVAE [28] and MolGAN [29]. We also performed an ablation study on our method to investigate the efficacy of reinforcement learning and auxiliary property prediction. We evaluated two ablation models, denoted A1 and A2. For model A1, we removed both the reward and predictor networks by setting $\beta _1=0$ and $\beta _2=0$, which indicates that the reinforcement learning and auxiliary property prediction were excluded from the learning objective. For model A2, the predictor network was removed by setting $\beta _1=1$ and $\beta _2=0$ to discard auxiliary property prediction only.

The performance of each model for molecular graph generation was evaluated with regard to three metrics: *Validity*, *Uniqueness*, and *Novelty*. We sampled 10,000 molecular graphs from each model. Then, *Validity* was calculated as the fraction of valid molecular graphs out of 10,000 generated molecular graphs. *Uniqueness* was the fraction of valid graphs that were not duplicates. *Novelty* was the fraction of valid graphs that were not included in the training dataset. The values of each metric ranged from 0 to 1. Because the primary goal is to generate molecular graphs that are valid, unique, and novel, we calculated the geometric mean (G-mean) of the three metrics for an overall comparison among the models.

All the experiments were implemented based on GPU-accelerated TensorFlow in Python.

### Results

**Fig. 2:**
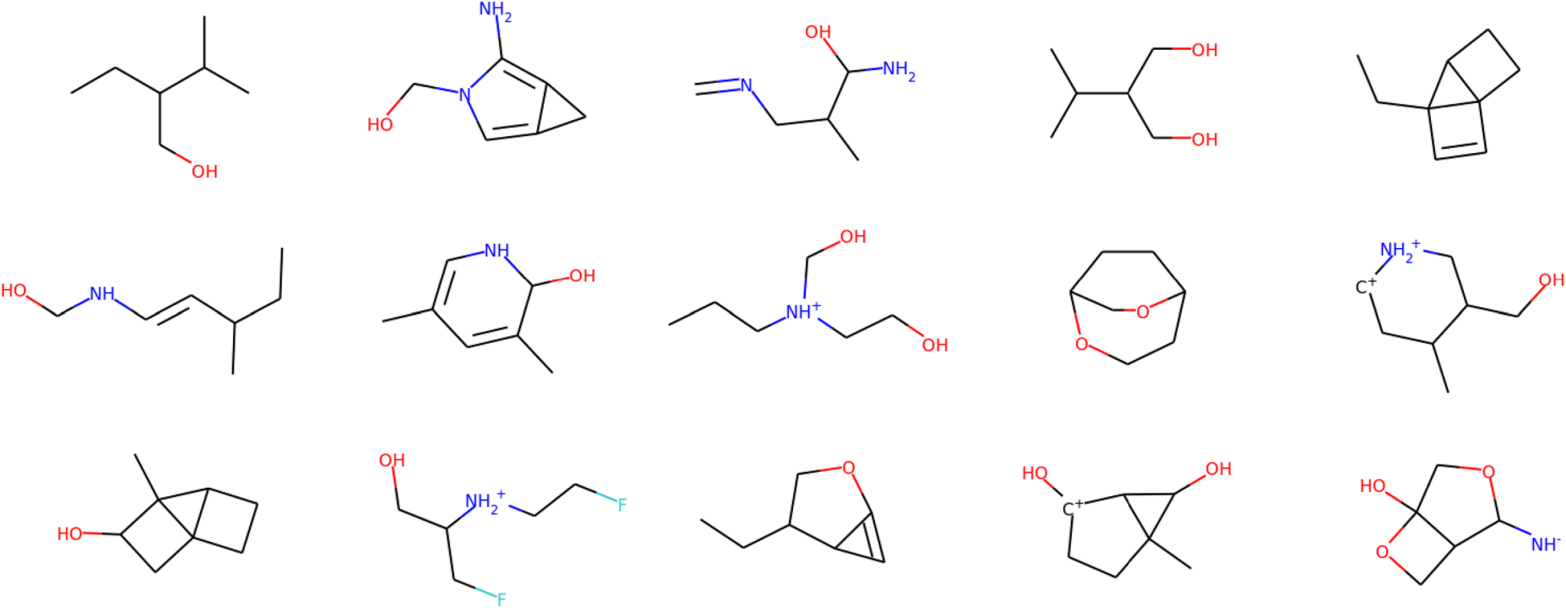
Examples of newly generated molecular graphs from QM9

**Fig. 3:**
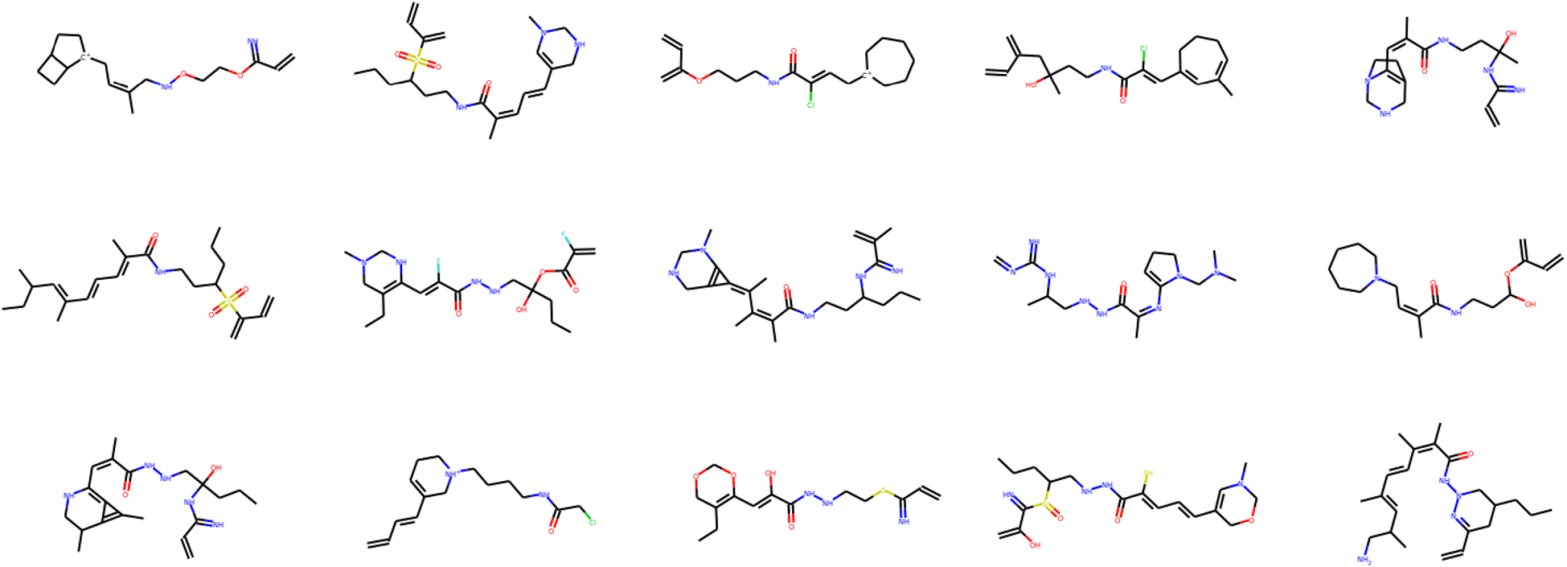
Examples of newly generated molecular graphs from ZINC

Table 1 presents the results of unconditional molecular graph generation for QM9 and ZINC. We report the validity, uniqueness, novelty, and G-mean for the five compared models. The best value in each row is highlighted in italics. Figures 2 and 3 show example molecular graphs generated randomly by the proposed models trained with QM9 and ZINC, respectively.

The proposed model exhibited the highest G-mean for both the QM9 and ZINC datasets, as the model achieved good performance for all three metrics. The proposed model exhibited a validity score of > 90% for both datasets, indicating that it almost always generated chemically valid molecules. Additionally, it generated more diverse molecules, as evident from its comparable uniqueness and novelty scores. In the case of ZINC, all the compared models yielded novelty of 1, indicating that all the generated molecules were not included in the training set. The baseline models yielded relatively good performance for QM9 but rarely generated valid molecular graphs for ZINC.

Compared with the ablated models, reinforcement learning helped the proposed model generate chemically valid molecules, as evident from the observation that model A2 was superior to model A1 in terms of validity. The auxiliary property prediction contributed to the diversification of the generated molecules, as the proposed model exhibited higher uniqueness than model A2.

**Table 1:** Performance comparison for unconditional molecular graph generation

Dataset	Metric	GraphVAE [28]	MolGAN [29]	Proposed		
				A1	A2	Full
QM9	*Validity*	0.610	*0.981*	0.550	0.965	0.945
	Uniqueness	*0.409*	0.104	0.293	0.275	0.343
	*Novelty*	0.850	*0.942*	0.612	0.737	0.806
	G-mean	0.596	0.458	0.462	0.581	*0.639*
ZINC	*Validity*	0.140	0.017	0.008	*0.926*	0.919
	Uniqueness	0.316	0.201	*0.949*	0.614	0.762
	*Novelty*	*1.000*	*1.000*	*1.000*	*1.000*	*1.000*
	G-mean	0.354	0.151	0.197	0.828	*0.888*

Best score for each metric is given in italic

Table 2 presents summary statistics for the newly generated molecules of each target condition by the proposed model. For conditional generation, the target conditions for MolWt and LogP were set as $\{120, 125, 130\}$ and $\{-\,0.4, 0.2, 0.8\}$, respectively, for QM9, and $\{300, 350, 400\}$ and $\{1.5, 2.5, 3.5\}$, respectively, for ZINC. The property distributions obtained via unconditional generation were similar to those of the training set. Compared with the unconditional generation, the conditional generation results exhibited a slightly lower G-mean and a smaller number of unique molecular graphs. As shown in Table 2, when a target condition was set, the proposed model successfully generated molecular graphs whose properties were close to the target value.

**Table 2:** Conditional molecular graph generation with proposed model

Dataset	Target condition	G-mean	Unique count	MolWt	LogP
QM9	Training set	–	100,000	122.97 ± 7.61	0.14 ± 1.16
	Unconditional generation	0.639	3243	123.01 ± 8.04	− 0.06 ± 1.36
	MolWt = 120	0.583	2316	121.85 ± 5.11	0.02 ± 1.36
	MolWt = 125	0.543	1947	125.11 ± 4.56	− 0.27 ± 1.22
	MolWt = 130	0.482	1475	128.98 ± 4.27	− 0.41 ± 1.33
	LogP = − 0.4	0.576	2399	122.97 ± 8.26	− 0.40 ± 0.73
	LogP = 0.2	0.543	2099	122.53 ± 8.17	0.19 ± 0.75
	LogP = 0.8	0.537	1989	122.17 ± 8.09	0.83 ± 0.72
ZINC	Training set	–	100,000	357.94 ± 65.48	2.62 ± 1.36
	Unconditional generation	0.888	7000	366.44 ± 51.63	2.49 ± 1.43
	MolWt = 300	0.742	4090	313.12 ± 13.72	1.91 ± 1.50
	MolWt = 350	0.796	5045	356.22 ± 12.66	2.24 ± 1.36
	MolWt = 400	0.805	5212	400.95 ± 13.66	2.78 ± 1.30
	LogP = 1.5	0.865	6470	352.33 ± 46.78	1.66 ± 0.94
	LogP = 2.5	0.860	6356	366.64 ± 48.30	2.46 ± 0.92
	LogP = 3.5	0.827	5658	381.96 ± 48.46	3.22 ± 0.85

### GuacaMol benchmarks

We further investigated the effectiveness of the proposed method with the distribution-learning benchmarks in the GuacaMol framework [41]. The benchmarks are based on a standardized subset of the ChEMBL database [42]. As the training set for the proposed model, we used molecules with up to 50 heavy atoms from the original dataset. We trained the model for 20 epochs with the same experimental settings as before. Then, we evaluated *Validity*, *Uniqueness*, and *Novelty* of generated molecular graphs by the model. In addition, whether the model is able to reproduce the distribution of the training set was assessed with *Kullback-Leibler Divergence (KLD)* and *Fréchet ChemNet Distance (FCD)*. For baselines, four SMILES generation models (LSTM, VAE, AAE, and ORGAN) and one molecular graph generation model (GraphMCTS) were compared, as implemented in [41].

The results for the distribution-learning benchmarks are shown in Table 3. Compared with the baseline models, the proposed model exhibited comparable or higher scores on validity, uniqueness, and novelty metrics. However, the proposed model yielded relatively lower KLD and FCD scores like GraphMCTS, which indicates that the proposed model was inferior on reproducing the underlying property distributions of the training set. Overall, the molecular graph generation models were very useful in generating chemically valid and diverse molecular graphs, while they were inferior to the SMILES generation models in distribution-learning.

**Table 3:** Results of GuacaMol distribution-learning benchmarks

Metric	SMILES-based				Graph-based	
	LSTM	VAE	AAE	ORGAN	GraphMCTS	Proposed
*Validity*	0.959	0.870	0.822	0.379	1.000	0.830
*Uniqueness*	1.000	0.999	1.000	0.841	1.000	0.944
*Novelty*	0.912	0.974	0.998	0.687	0.994	1.000
*KLD*	0.991	0.982	0.886	0.267	0.522	0.554
*FCD*	0.913	0.863	0.529	0.000	0.015	0.016

## Conclusion

While the non-autoregressive approach has the advantage of fast and computationally efficient generation of molecular graphs without any iterative procedure, the existing methods suffer from low performance. To overcome this limitation, we proposed an efficient learning method to build a graph VAE that generates molecular graphs in a non-autoregressive manner. In order to improve the generation performance, we introduced approximate graph matching, reinforcement learning, and auxiliary property prediction into the training of the model. Experimental validation using QM9 and ZINC datasets demonstrated the effectiveness of the proposed method. Compared with existing methods, the proposed method exhibited higher performance for molecular graph generation with a validity score of > 90% as well as improved diversity of the generated molecular graphs for both datasets. We also demonstrated that the proposed model can conditionally generate novel molecular graphs satisfying specified target conditions.

We believe that the proposed method can serve as an efficient tool for the discovery of new materials. Successful application of the method will allow automatic design of desired chemical structures with targeted conditions by learning implicit knowledge from data without explicit knowledge from human experts, thereby meriting further investigations. The generated molecular graphs can be examined further to obtain realistic chemical structures with desired properties.

One downside of the proposed method its high complexity with regard to space and time. Both the space and time complexity increase with the size of the graphs, owing to the use of graph neural networks. Thus, the proposed method would be impractical for learning and generating large molecular graphs, e.g., hundreds of heavy atoms in a molecule. In the future, research will be performed to reduce the complexity for generating larger molecular graphs efficiently.

## Data Availability

The source code and datasets used in this study are available online at http://github.com/seokhokang/graphvae_approx/. The original QM9 and ZINC data are publicly accessible from http://quantum-machine.org/datasets/ and http://zinc15.docking.org/, respectively.
